# Pelvic Floor Muscle Training on Stress Urinary Incontinence in Power- and Weightlifters: a Pilot Study

**DOI:** 10.1007/s00192-024-05801-8

**Published:** 2024-05-17

**Authors:** Kari Bø, Ragnhild Haug Lillegård, Kristina Lindquist Skaug

**Affiliations:** 1 Department of Sports Medicine, Norwegian School of Sport Sciences, Oslo, Norway; 2 Department of Obstetrics and Gynecology, Akershus University Hospital, Lørenskog, Norway

**Keywords:** Female athlete, Pelvic floor muscle training, Powerlifter, Stress urinary incontinence, Weightlifters, Women’s health

## Abstract

**Introduction and hypothesis:**

Stress urinary incontinence (SUI) is defined as involuntary leakage of urine on physical effort and is prevalent among power- and weightlifters. However, there is scant knowledge on treatment options for this population. The aim of this pilot study was to evaluate the potential outcomes and feasibility of a pelvic floor muscle training (PFMT) program on SUI in nulliparous female power- and weightlifters.

**Methods:**

This was a case-series study, including one weightlifter and two powerlifters aged 21–32 years. The participants conducted 12 weeks of PFMT at home, with weekly follow-up by a physiotherapist. Change in total score of the International Consensus of Incontinence Questionnaire Urinary Incontinence Short Form (ICIQ-UI-SF) was the primary outcome. Secondary outcome was perceived change assessed by the Patient Global Impression of Improvement (PGI-I) Scale and impact on sport participation. PFM strength, endurance, and resting pressure was measured using vaginal manometry. Feasibility was evaluated as adherence to training and self-efficacy (Self Efficacy Scale for Practicing Pelvic Floor Exercises).

**Results:**

One athlete reduced their ICIQ-UI-SF score and experienced improvement in symptoms. One athlete reported no change, and one reported a worsening of symptoms. All three participants improved PFM strength and endurance, completed the testing, and 12 weeks of PFMT, but adherence varied between 40 and 80%. Participants reported a lack of time and energy and forgetting to perform the exercises, as reasons for low adherence.

**Conclusion:**

There were varying effects of a 12-week PFMT program on SUI in three strength athletes. The results can create the basis for a future randomized controlled trial.

## Introduction

Urinary incontinence (UI) is defined by the International Continence Society (ICS) as “complaint of involuntary loss of urine” [1]. The most common form of UI in women is stress urinary incontinence (SUI), defined as the “complaint of involuntary loss of urine on effort or physical exertion (e.g., sporting activities), or on sneezing or coughing” [1]. Urgency urinary incontinence (UUI) is the complaint of involuntary leakage accompanied by or immediately preceded by urgency and mixed urinary incontinence (MUI) is a combination of the two [1]. High prevalence rates of UI among both parous and nulliparous female athletes and exercisers have been reported in several cross-sectional studies [2–4]. The highest prevalence rates are seen in high impact sports, such as running, gymnastics, ball games, and trampoline jumping [3, 5]. Recent prevalence studies among power- and weightlifters have reported a prevalence of UI between 32 and 50% [6–8].

High-quality randomized controlled trials (RCTs) and meta-analyses have demonstrated the effectiveness of pelvic floor muscle training (PFMT) for SUI and MUI in the general female population [9, 10]. As strength training of the pelvic floor muscles (PFM) has no side effects, there is international consensus that PFMT should be first-line treatment for these conditions [10]. However, there is limited knowledge about the effects of PFMT in physically active women [4], and no studies have been found evaluating PFMT for UI in strength sports [11, 12]. Women participating in strength sports differ from populations studied previously, as they frequently expose the pelvic floor to high loads owing to increases in intra-abdominal pressure during lifting of heavy external loads [4, 6]. It has therefore been suggested that treating these athletes with PFMT might be more challenging than in the general female population. On the other hand, one could also argue that elite athletes, and especially strength athletes, are used to performing regular strength training and hence, adding PFMT to their daily routines could be feasible [4].

As far as we have ascertained, there are no clinical trials evaluating the effect of PFMT for treating UI in female strength athletes and, therefore, the aims of the present pilot study were to assess the effects and feasibility of PFMT on SUI in a case series of female nulliparous power- and weightlifters.

## Materials and Methods

### Design

This was a case-series pilot study, approved by the regional ethics committee (499117/REK Sør-øst B, 30.10.2022) and the Norwegian Centre for Research Data (NSD: 416437, 14.10.2022). All participants gave electronic and written informed consent before participation. The Case Report guidelines (CARE) were followed in reporting the study [13].

### Participants

Participants were recruited through the Norwegian powerlifting and weightlifting federations, local clubs, trainers, and social media (Facebook, Instagram) between October and December 2022. Inclusion criteria were being nulliparous, aged ≥ 18 years, and actively engaged in weightlifting and/or powerlifting (training ≥ 3 days per week and participating in competitions, no specific level) with self-reported SUI. SUI was verified by the International Consultation on Incontinence Questionnaire Urinary Incontinence Short Form (ICIQ-UI-SF), where a sum-score of ≥ 3 was required to be included. Furthermore, the participants had to answer, “I leak when I am physical active/exercising” to the ICIQ-UI-SF question “When does urine leak?” to be eligible. Exclusion criteria were previous surgery to the pelvic region, ongoing pregnancy or planned pregnancy during the intervention period, previous musculoskeletal injury within the past 6 months, ongoing injury that would negatively impact participation in training, medical conditions or disorders, and use of medication that may negatively affect the pelvic floor. Eligible participants were invited to participate in two test sessions (at baseline and post-intervention) at the university.

### Intervention

The intervention consisted of daily individual strength training of the PFM for 12 weeks, approximately 10 min per day. This was a home-based training program with weekly follow-ups by a physiotherapist by phone or in-person. The training did not require any equipment. Prior to the intervention, the participants were taught about PFM anatomy and muscle function, exercise physiology, and the purpose of the training. Ability to perform a correct PFM contraction was assessed by vaginal palpation [14–16]. Initially, the training included three sets of 8–12 maximum PFM contractions held for 6–8 s each day, but the repetition count and duration of contractions were individually customized based on the PFM testing. Training progression was individually tailored throughout the intervention period, with options to change positions, increase the number of contractions, and to add 3–4 fast contractions on top of each holding period [17–19]. The participants also received a booklet with information about the training program and progression. More details of the intervention are given in the Consensus of Exercise Reporting Template as supplementary information.

### Outcomes

#### Primary Outcome: ICIQ-UI-SF

The primary outcome was change in total score of the ICIQ-UI-SF [20]. The ICIQ-UI-SF is a reliable and valid instrument that evaluates the frequency of leakage, amount of leakage, impact on daily life, and type of urinary incontinence [20].

The questionnaire comprises four questions, with three of them contributing to a total score ranging from 0 to 21, whereas the fourth question categorizes the type of UI. A minimal important difference of 2.5 in the ICIQ-UI-SF total score has been established [21].

#### Patient Global Impression of Improvement Scale

The participants were asked to rate their perceived change in UI post-test, using a validated seven-point scale. The response choices ranged from “very much better” to “very much worse” [22].

#### Participation in Sports

The following questions were added regarding how UI affects participation in sports [23, 24]:

- "To what extent does urinary leakage affect your well-being or participation in sport activities?" The participants responded on a numeric rating scale from 0 = not at all to 10 = a lot.
- "Do you ever avoid exercise, physical activity, and/or specific exercises because you are concerned about leaking urine?" Response alternatives were from 0 = never to 3 = very often. The responses to these two questions were summarized in a scale from 0 to 13.
- "If you leak urine during physical activity, exercise, and/or competition—how does this affect you?" Response alternatives were: not at all, I lose concentration, I am afraid the leakage will be visible, I am afraid of odor, I am afraid of doing erroneous movements, I am frustrated/irritated, I am embarrassed, I am afraid it will happen again, the leakage negatively affects sport performance. The participants could respond to more options.

#### Manometry Measurements of the PFM

A high-precision pressure transducer connected to a vaginal balloon catheter (Camtech AS, Sandvika, Norway) was used to measure vaginal resting pressure, PFM strength (maximum voluntary contraction), and PFM endurance (10-s hold of maximum contraction) [25, 26]. Vaginal resting pressure (cmH_2_O) was measured as the difference between atmospheric pressure and the vaginal high-pressure area at rest, without any voluntary activity in the PFMs. Muscle strength (cmH_2_O) was calculated as the average of three maximum voluntary contractions. Endurance was defined as a sustained maximum contraction and was measured during the first 10 s as the area under the curve (cmH_2_O/s) [27]. The instructions given to the participants were standardized [26], and the method has been found to have good intra- and interrater reliability [25–27]. Before the manometer measurement, ability to perform a correct PFM contraction was assessed by visual observation and vaginal palpation by a trained physical therapist. All assessments were done in a crock-lying supine position. To ensure valid PFM contractions during manometry, only measurements with simultaneous observable inward movement of the catheter/perineum were recorded [14]. If the participants were unable to contract, manual techniques such as fast stretch, tapping, and lateral pressure, in addition to more verbal information, were used to explain how to perform a correct PFM contraction. Strength of the contraction and ability to hold created the basis for the starting point and follow-up of the individually prescribed PFMT program.

### Feasibility Aspects

Using the reliable Self-Efficacy Scale for Practicing Pelvic Floor Exercises (SESPPFE), the participants were asked to rate their self-efficacy for PFMT before the commencement of the training period and after 1 month of training [28]. The scale includes 17 items, and the score ranged from 0 to 100, where a higher score indicates higher self-efficacy for PFMT. Cronbach’s alpha is 0.92, and the instrument can differentiate between women who adhere and those do not adhere to PFMT prescription [28, 29].

#### Adherence

A training diary was used to assess adherence to the training program. The participants were instructed to record all completed training sessions involving PFMT. Any uncompleted training sessions were also logged, along with a report of reasons for non-adherence. When calculating adherence to the training, only sessions completed as prescribed were included (number of days where the training consisted of three sets of PFM contractions).

#### Participants' Perspective on the PFMT Program

Through open-ended questions, participants were asked to provide feedback on the training program. The question "Lastly, we ask you to briefly describe (1–3 sentences) your experience with the training program" was distributed after the intervention period. At the post-test, the participants were also asked two standardized questions: "How did you experience the PFMT?" and "What were the main reasons for not adhering to the training protocol?"

### Data Collection and Procedures

The study was conducted between November 2022 and March 2023. One week prior to the baseline evaluation, informed consent, background variables (age, education, body mass index, training background, training habits, chronic diseases, medicines, knowledge about the PFM and PFMT), the ICIQ-UI-SF, whether UI affected sport performance, and SESPPFE were collected in an electronic questionnaire (Survey Xact). The baseline clinical evaluation included measurement of the PFM, weight, and height. All participants were reassessed after the 12-week intervention period, including the same electronic questionnaire (ICIQ-UI-SF, PGI-I and whether UI affected sport performance) and manometry assessment of vaginal resting pressure, PFM strength, and endurance. In addition, data on adherence and perspectives on training were assessed.

### Data Analyses

Background variables are reported for each participant. Total score on ICIQ-UI-SF and other outcomes are reported for each individual participant with pre- and post-scores, as well as the change and direction of change. Adherence was calculated as percentage of completed training days and was reported for each participant.

## Results

Five athletes contacted the research team, three of whom fulfilled the inclusion criteria and agreed to participate after receiving detailed information: one weightlifter and two powerlifters. Background characteristics are presented in Table 1. All athletes reported both SUI and UUI.

**Table 1:** Background variables of three female strength athletes (participants 1, 2, and 3)

	Participant 1	Participant 2	Participant 3
Age	22	21	32
Sport	Power lifting	Power lifting	Weightlifting
Education	Student at university	Student at university	University ≥ 4 years
BMI kg/m^2^	24.8	23.6	23.1
Years of sport participation	1 year and 6 months	3 years and 1 month	2 years and 10 months
Training hours/week in sport	14	10	8
Other training sessions	No	Cardiovascular	No
		Flexibility	

### Outcome of UI and PFM Variables

#### ICIQ-UI-SF Total Score

Table 2 presents the results related to the outcome of the intervention. Participant 1 reduced her total score on the ICIQ-UI-SF by more than the minimum clinically significant change of 2.5 from pre- to post-test. Participant 2 improved, but not more than the minimum clinically significant change, and participant 3 reported to be worse.

**Table 2:** International Consultation on Incontinence Questionnaire Urinary Incontinence Short Form (*ICIQ-UI-SF*), Patient Global Impression of Improvement (*PGI-I*) and how much urinary incontinence affects sport performance before and after 12 weeks of pelvic floor muscle training in three female strength athletes

	Participant	Pre-test	Post-test	Change
ICIQ-UI-SF total score (0–22)				
	1	13	6	−7^a^
	2	6	6	0
	3	9	8	−1
Frequency of leakage				
	1	3	1	−2
	2	2	2	0
	3	2	1	−1
Amount of leakage				
	1	2	2	0
	2	2	2	0
	3	2	4	+2
Affecting activities of daily living				
	1	8	3	−5
	2	3	3	0
	3	5	3	−2
PGI-I				
	1		A little better	
	2		No change	
	3		A little worse	
Affecting sport performance (0–13)				
	1	5	4	−1
	2	1	1	0
	3	3	5	+2

^a^Change above the minimum detectable difference

The results of the ICIQ-UI-SF corresponded with the responses of the PGI-I (Table 2).

#### Participation in Sports

Before the training intervention, all participants reported that UI affected their participation in sports. After 12 weeks of PFMT, participant 1 improved her score by one point, participant 2 reported no change, and participant 3’s score worsened three points (Table 2).

#### Manometry Measurements of the PFM

At pre-test all participants were able to perform a correct PFM contraction assessed by vaginal palpation after individual instruction. Participant 3 needed thorough teaching before correct contraction was established and had a weak contraction (Table 3).

**Table 3:** Vaginal resting pressure, pelvic floor muscle (*PFM*) strength, and PFM endurance before and after 12 weeks of pelvic floor muscle training in three female strength athletes

	Participant	Pre-test	Post-test	Change
Vaginal resting pressure (cmH_2_O)				
	1	30.6	24.9	−5.7
	2	35.5	34.7	−0.8
	3	31.2	34.4	+ 3.2
PFM strength (cmH_2_O)				
	1	10.3	23.5	+ 13.2^a^
	2	29.6	30.1	+ 0.5
	3	5.7	8.2	+ 2.5
PFM endurance (holding time; cmH_2_/10 s)				
	1	67	107	+ 40
	2	143	195	+ 52
	3	28	55	+ 27

^a^Change above minimal detectable value

Vaginal resting pressure decreased in two participants and increased in one. All three participants increased their PFM strength and endurance. The only measurement that exceeded the smallest detectable change of the measuring instrument was the PFM strength of participant 1.

### Feasibility

#### Self-Efficacy Scale for Practicing Pelvic Floor Exercises

Table 4 presents the SESPPFE scores at baseline and after 1 month. After 1 month, participants 1 and 3 reduced the self-efficacy score by 3.8 and 8.2 points respectively. Participant 2 increased their score by 1.7.

**Table 4:** Feasibility aspects with pelvic floor muscle training in female strength athletes (n = 3)

	Participant	Pre-test	At one month	Adherence was reported throuoghout the training period
PFSES (0–100)				
	1	88.2	84.4	
	2	61.2	62.9	
	3	84.7	76.5	
Adherence to prescribed protocol (% of total 84 sessions)				
	1			40.5%
	2			70.2%
	3			80.1%
Mean number of PFM exercise sessions /week				
	1			3
	2			5
	3			6
Mean number of PFM contractions per session				
	1			30
	2			24
	3			18

*PFSES *Pelvic Floor Self-Efficacy Scale

*PFM *Pelvic floor muscle

#### Adherence

Adherence to the training protocol is shown in Table 4. Adherence varied between 40 and 80%. All participants reported performing three or more PFMT sessions per week when exercising, and the number of contractions within each session varied between 18 and 30.

#### The Participants' Perspective on Training

The participants’ experience and perspective on the PFMT showed that it was easy and motivating to do the PFMT program at the beginning, but it became more challenging to maintain daily exercises over time. Reasons for not adhering to the program were lack of time, lack of energy, illnesses, and that they forgot to do the exercises. One participant also reported that it was demotivating when no improvement was experienced. The participants reported that the training needed substantial concentration. The participant who reported being worse stated that she had become more aware of her leakage and had increased the sport training workload during the intervention period, and that this might have affected her outcome. No adverse effects were reported.

All participants utilized the pre-contraction technique prior to coughing and sneezing ("the Knack") [30]. Two participants eliminated leakage during coughing/sneezing after the training intervention by use of this technique, and one participant also utilized the technique during heavy lifts, particularly deadlifts, believing that it provided better core support during the lift.

## Discussion

This case series of three strength athletes conducting 12 weeks of PFMT for UI found varying outcomes among the participants. The individual results of the primary outcome variable, ICIQ-UI-SF concurred with the results of the PGI-I. PFM strength, and endurance increased in all participants. The participants completed the intervention and pre- and post-test testing, but adherence varied between 40 and 80%. Participants reported a lack of time and energy and forgetting to perform the exercises as reasons for low adherence.

Case studies and case series cannot establish causal inference and effect sizes. However, such designs can be used as a pilot to establish preliminary outcome data and important knowledge for planning interventions of future robust and high-quality RCTs [31]. PFMT has level 1, recommendation A, to be first-line treatment for SUI and MUI in the general female population, with cure rates reaching 80% [9, 10]. However, no studies have been found in female strength athletes [11, 12] and the results of this case series can therefore indicate the outcome of the training program. Although our inclusion criterion was SUI, all three participants also reported UUI, meaning that they have MUI. PFMT has been shown to most effective in women with SUI only, and may be one of other factors explaining the modest improvement found in our study [9]. Three pre-/post-test design studies such as the present study have been published concluding with a positive outcome [32–34]. None of these studies included strength athletes. Da Roza et al. [33] assessed seven sport students of gymnastics, trampoline, figure skating, synchronized swimming, and handball, and reported a reduction in ICIQ-UI-SF from mean 4.08 (SD 6.0) to 0.75 (SD 2.6), *p* = 0.027. Only one full-scale RCT has been found assessing the effect of PFMT in athletes. Ferreira et al. [35] randomized 32 female volleyball players to 3 months of PFMT or control and reported statistically significant results using the pad test and frequency of leakage and amount of leakage using the pad test. These results are promising, but future RCTs are needed to assess the effect of PFMT in strength athletes as their impact on the pelvic floor is different from sports consisting of running and jumping/landing.

As the above-mentioned studies use different measurement methods for assessing the independent variables (vaginal resting pressure, PFM strength, and endurance), their results cannot be directly compared with ours. The results of the present study showed that PFM strength and endurance were increased to similar levels, which has been found in studies of the general female population using the same apparatus [27]. However, the one participant reporting to be worse had no improvement, although she had high adherence, indicating that her contractions may not have been effective in increasing urethral closure pressure [36]. She was also the participant who had difficulties contracting correctly and there may also have been other factors such as change in exercise routines with increasing loads during the training period that may have affected her negatively. One could also speculate that she had an increase in UUI with improper PFMT that either was bearing down or was not adequately relaxing in between. Interestingly, all participants performed “the Knack” (consciously contracted the PFM before an increase in intra-abdominal pressure) and this was reported to be effective for all. Similar results have been shown in the general female population during coughing and activities of daily living including inhibiting the urge to void and UUI [30]. This technique may therefore have an immediate preventive effect on UI among strength athletes and should be investigated in future RCTs.

One cannot assume that athletes have knowledge about the PFM and PFMT [6, 23, 24, 37]. A recent study found that a minority of athletes were able to perform correct PFM contraction at their first assessment [38]. A common error in attempts at PFM contraction is to strain (pushing downward) instead of performing a correct PFM contraction (squeezing around the pelvic openings and lifting the pelvic floor forward and cranially) [36]. Thorough instruction and information about the pelvic floor and its function is mandatory before commencing a PFMT intervention, and so is feedback of the participants’ ability to perform a correct and strong contraction. Dos Santos et al. [38] reported that all athletes in their study who were unable to contract at their first visit, learned how to perform the exercise after individual instruction and assessment by a physiotherapist. In the present study there was no monitoring of the PFM contractions during the intervention period and one of the athletes had challenges in performing a correct and strong PFM contraction. Closer individual supervision and follow-up of her attempts to contract might have improved her outcome. However, not all women succeed with PFMT and thus, the three participants of the present pilot study may mirror a larger sample of women performing PFMT to treat SUI [9].

Feasibility was assessed using the SESPPFE, adherence, and three open questions on how the participants experienced PFMT. We have not been able to find other studies reporting on self-efficacy for PFMT in athletes. Sacomori et al. [28] found that three factors—performance expectation considering the action, performance expectation considering the preparation for action, and outcome expectations—accounted for 65.3% of the total variance in women from the general community and in a postpartum population. Applying hierarchical regression analyses incorporating treatment group, age, education, disease-related factors (severity of UI; pelvic floor muscle strength; BMI), outcome expectations, and self-efficacy in 72 women, of whom 43 performed everyday PFMT, showed that only baseline self-efficacy predicted adherence. We agree that self-efficacy is important to include in future studies of PFMT. In the present study the SESPPFE was stable within the first month of PFMT in two of the participants but decreased in the participant who reported to be slightly worse. The SESPPFE concurred with the response to the open questions on how they perceived the training.

Our results finding that PFMT needs concentration, is easy to forget, and can be difficult to prioritize in a busy schedule among female athletes corresponds with results of adherence challenges in the general population [39]. Although the athletes in general have high motivation for regular and intensive training, they spend a considerable part of their leisure time on their sports and may therefore have less tolerance and time than others for extra tasks such as PFMT. Including PFMT as a natural part of their regular training session could be a way of increasing adherence. In an epidemiological study on powerlifters and Olympic weightlifters, Skaug et al. [6] found that 20.6% of the women and 58.8% of the men had never heard of PFMs. More than 40% of the women and 70% of the men did not know why and how to train the PFMs. However, almost 80% of the women and half of the men responded that they would do PFMT to prevent or treat pelvic floor disorders if they knew how to perform the exercises. Nevertheless, although motivated for general strength training, the athletes may consider PFMT less motivating than other exercises.

To date, PFMT does not seem to be included in athletic strength training protocols, and coaches may not know how to teach effective PFMT. RCTs are needed to investigate whether including PFMT as part of regular training in athletes can improve UI. RCTs in the general population have shown that PFMT is more effective after supervised training and a closer follow-up with clinical assessment of development of PFM strength may be important for motivation [9, 10].

Strengths of the present study are the use of reliable and valid outcome measures, for registration of UI, measurement of PFM variables, self-efficacy, and adherence. The PFMT protocol followed recommendations for general strength training, and the program has been used in several RCTs, showing a statistically and clinically meaningful effect on female SUI [17–19]. A comprehensive pool of outcome measures for feasibility factors combining both quantitative and qualitative data was utilized, giving important information for planning a future RCT among strength athletes. The limitation of the study is the research design, as no causal inference can be drawn from a case series, and the results therefore can only indicate a direction of the effect. In addition, in the present study, three possible outcomes of an intervention was found: some effect, no effect, and some worsening of UI; thus, no convincing direction of a possible effect can be seen. However, a preliminary power-calculation for a future RCT can be conducted based on the present results. The average change in the primary outcome measure, total score on the ICIQ-UI-SF, from pre-test to post-test for all three participants, was calculated to be −2.67 (SD: 3.09). Based on these values, and 0 change in the control group (SD: 3.09), with 80% power and a significance level set at 0.05, a total of 22 participants in each group would be needed. To account for uncertainty in the power calculation, as well as potential dropouts from the study (20%), we would advise including a total of 70 participants [40].

## Conclusion

In this pilot study, we found varying effects of a 12-week PFMT program on three female strength athletes with UI. All athletes improved PFM strength and endurance. Adherence to the training program varied between 40 and 80% and concurred with the outcome. The results of the pilot can guide future randomized controlled trials of PFMT for UI in strength athletes.

## References

[1] Haylen BT, de Ridder D, Freeman RM (2010). An International Urogynecological Association (IUGA)/International Continence Society (ICS) joint report on the terminology for female pelvic floor dysfunction. Neurourol Urodyn.

[2] Teixeira RV, Colla C, Sbruzzi G, Mallmann A, Laureano Paiva L (2018). Prevalence of urinary incontinence in female athletes: a systematic review with meta-analysis. Int Urogynecol J.

[3] de Mattos Lourenco TR, Matsuoka PK, Baracat EC, Haddad JM (2018). Urinary incontinence in female athletes: a systematic review. Int Urogynecol J.

[4] Bo K, Nygaard IE (2020). Is physical activity good or bad for the female pelvic floor? A narrative review. Sports Med.

[5] Nygaard IE, Shaw JM (2016). Physical activity and the pelvic floor. Am J Obstet Gynecol.

[6] Skaug KL, Engh ME, Frawley H, Bo K (2022). Prevalence of pelvic floor dysfunction, bother, and risk factors and knowledge of the pelvic floor muscles in Norwegian male and female powerlifters and Olympic weightlifters. J Strength Cond Res.

[7] Wikander L, Kirshbaum MN, Waheed N, Gahreman DE (2022). Urinary incontinence in competitive women weightlifters. J Strength Cond Res.

[8] Wikander L, Kirshbaum MN, Waheed N, Gahreman DE (2021). Urinary incontinence in competitive women powerlifters: a cross-sectional survey. Sports Med Open.

[9] Dumoulin C, Cacciari LP, Hay-Smith EJC (2018). Pelvic floor muscle training versus no treatment, or inactive control treatments, for urinary incontinence in women. Cochrane Database Syst Rev.

[10] Dumoulin C, Booth J, Cacciari L, et al. Conservative management of UI and POP in adults, including neurological patients Chapter 8. In: Cardozo L, Rovner E, Wagg A, Wein A, Abrams P, editors. Incontinence. pp. 795–1038.

[11] Fukuda FS, Arbieto ERM, Da Roza T, Luz S (2023). Pelvic floor muscle training in women practicing high-impact sports: a systematic review. Int J Sports Med.

[12] Romero-Franco N, Molina-Mula J, Bosch-Donate E, Casado A (2021). Therapeutic exercise to improve pelvic floor muscle function in a female sporting population: a systematic review and meta-analysis. Physiotherapy.

[13] Riley DS, Barber MS, Kienle GS (2017). CARE guidelines for case reports: explanation and elaboration document. J Clin Epidemiol.

[14] Bø K, Kvarstein B, Hagen R, Larsen S (1990). Pelvic floor muscle exercise for the treatment of female stress urinary incontinence. II. Validity of vaginal pressure measurements of pelvic floor muscle strength and the necessity of supplementary methods for control of correct contraction. Neurourol Urodyn.

[15] Frawley H, Shelly B, Morin M (2021). An International Continence Society (ICS) report on the terminology for pelvic floor muscle assessment. Neurourol Urodyn.

[16] Kegel AH (1948). Progressive resistance exercise in the functional restoration of the perineal muscles. Am J Obstet Gynecol.

[17] Bø K, Hagen RH, Kvarstein B (1990). Pelvic floor exercise for the treatment of female stress urinary incontinence. III. Effects of two different degrees of pelvic floor muscle exercises. Neurourol Urodyn.

[18] Bo K, Talseth T, Holme I (1999). Single blind, randomised controlled trial of pelvic floor exercises, electrical stimulation, vaginal cones, and no treatment in management of genuine stress incontinence in women. BMJ.

[19] Braekken IH, Majida M, Engh ME, Bo K (2010). Can pelvic floor muscle training reverse pelvic organ prolapse and reduce prolapse symptoms? An assessor-blinded, randomized, controlled trial. Am J Obstet Gynecol.

[20] Avery K, Donovan J, Peters TJ, Shaw C, Gotoh M, Abrams P (2004). ICIQ: a brief and robust measure for evaluating the symptoms and impact of urinary incontinence. Neurourol Urodyn.

[21] Nystrom E, Sjostrom M, Stenlund H, Samuelsson E (2015). ICIQ symptom and quality of life instruments measure clinically relevant improvements in women with stress urinary incontinence. Neurourol Urodyn.

[22] Yalcin I, Bump RC (2003). Validation of two global impression questionnaires for incontinence. Am J Obstet Gynecol.

[23] Gram MCD, Bo K (2020). High level rhythmic gymnasts and urinary incontinence: prevalence, risk factors, and influence on performance. Scand J Med Sci Sports.

[24] Skaug KL, Engh ME, Frawley H, Bo K (2021). Urinary and anal incontinence among female gymnasts and cheerleaders—bother and associated factors. A cross-sectional study. Int Urogynecol J.

[25] Tennfjord MK, Engh ME, Bo K (2017). An intra- and interrater reliability and agreement study of vaginal resting pressure, pelvic floor muscle strength, and muscular endurance using a manometer. Int Urogynecol J.

[26] Bø K, Kvarstein B, Hagen R, Larsen S (1990). Pelvic floor muscle exercise for the treatment of female stress urinary incontinence. I. Reliability of vaginal pressure measurements of pelvic floor muscle strength. Neurourol Urodyn.

[27] Braekken IH, Stuge B, Tveter AT, Bo K (2021). Reliability, validity and responsiveness of pelvic floor muscle surface electromyography and manometry. Int Urogynecol J.

[28] Sacomori C, Cardoso FL, Porto IP, Negri NB (2013). The development and psychometric evaluation of a self-efficacy scale for practicing pelvic floor exercises. Braz J Phys Ther.

[29] Sacomori C, Berghmans B, de Bie R, Cardoso FL (2020). Predictors for adherence to a home-based pelvic floor muscle exercise program for treating female urinary incontinence in Brazil. Physiother Theory Pract.

[30] Miller JM, Hawthorne KM, Park L (2020). Self-perceived improvement in bladder health after viewing a novel tutorial on knack use: a randomized controlled trial pilot study. J Womens Health (Larchmt).

[31] Herbert R, Jamtvedt G, Mead J, Hagen KB (2005). Outcome measures measure outcomes, not effects of intervention. Aust J Physiother.

[32] Rivalta M, Sighinolfi MC, Micali S, de Stefani S, Torcasio F, Bianchi G (2010). Urinary incontinence and sport: first and preliminary experience with a combined pelvic floor rehabilitation program in three female athletes. Health Care Women Int.

[33] Da Roza T, Poli de Araujo M, Viana R (2012). Pelvic floor muscle training to improve urinary incontinence in young, nulliparous sport students: a pilot study. Int Urogynecol J.

[34] Sherman RA, Davis GD, Wong MF (1997). Behavioral treatment of exercise-induced urinary incontinence among female soldiers. Mil Med.

[35] Ferreira S, Ferreira M, Carvalhais A, Ribeiro Santos PC (2014). Reeducation of pelvic floor muscles in volleyball athletes. Rev Assoc Med Bras.

[36] Bump RC, Hurt WG, Fantl JA, Wyman JF (1991). Assessment of Kegel pelvic muscle exercise performance after brief verbal instruction. Am J Obstet Gynecol.

[37] Cardoso AMB, Lima C, Ferreira CWS (2018). Prevalence of urinary incontinence in high-impact sports athletes and their association with knowledge, attitude and practice about this dysfunction. Eur J Sport Sci.

[38] Dos Santos KM, Da Roza T, Mochizuki L, Arbieto ERM, Tonon da Luz SC (2019). Assessment of abdominal and pelvic floor muscle function among continent and incontinent athletes. Int Urogynecol J.

[39] Dumoulin C, Hay-Smith J, Frawley H (2015). 2014 consensus statement on improving pelvic floor muscle training adherence: International Continence Society 2011 State-of-the-Science Seminar. Neurourol Urodyn.

[40] Sealed Envelope. Power calculator for continuous outcome superiority trial. 2012 https://www.sealedenvelope.com/power/continuous-superiority/. Accessed 29 November 2023.

